# Future trends in environmental mercury concentrations: implications for prevention strategies

**DOI:** 10.1186/1476-069X-12-2

**Published:** 2013-01-07

**Authors:** Elsie M Sunderland, Noelle E Selin

**Affiliations:** 1 Department of Environmental Health, Harvard School of Public Health & School of Engineering and Applied Sciences, Harvard University, Boston, Massachusetts, USA; 2 Engineering Systems Division & Department of Earth, Atmospheric, and Planetary Sciences, Massachusetts Institute of Technology, Cambridge, Massachusetts, USA

**Keywords:** Methylmercury, Health, Wildlife, Fish, Exposures, Costs, Emissions, Risk

## Abstract

In their new paper, Bellanger and coauthors show substantial economic impacts to the EU from neurocognitive impairment associated with methylmercury (MeHg) exposures. The main source of MeHg exposure is seafood consumption, including many marine species harvested from the global oceans. Fish, birds and other wildlife are also susceptible to the impacts of MeHg and already exceed toxicological thresholds in vulnerable regions like the Arctic. Most future emissions scenarios project a growth or stabilization of anthropogenic mercury releases relative to present-day levels. At these emissions levels, inputs of mercury to ecosystems are expected to increase substantially in the future, in part due to growth in the legacy reservoirs of mercury in oceanic and terrestrial ecosystems. Seawater mercury concentration trajectories in areas such as the North Pacific Ocean that supply large quantities of marine fish to the global seafood market are projected to increase by more than 50% by 2050. Fish mercury levels and subsequent human and biological exposures are likely to also increase because production of MeHg in ocean ecosystems is driven by the supply of available inorganic mercury, among other factors. Analyses that only consider changes in primary anthropogenic emissions are likely to underestimate the severity of future deposition and concentration increases associated with growth in mercury reservoirs in the land and ocean. We therefore recommend that future policy analyses consider the fully coupled interactions among short and long-lived reservoirs of mercury in the atmosphere, ocean, and terrestrial ecosystems. Aggressive anthropogenic emission reductions are needed to reduce MeHg exposures and associated health impacts on humans and wildlife and protect the integrity of one of the last wild-food sources globally. In the near-term, public health advice on safe fish consumption choices such as smaller species, younger fish, and harvests from relatively unpolluted ecosystems is needed to minimize exposure risks.

## Background

In their recent article, Bellanger et al. [1] quantified the monetary benefits from control of methylmercury (MeHg) toxicity in European Union (EU) countries at between €8,000 and €9,000 million per year. The authors used population biomarker data to estimate that 1.5 to 2.0 million EU children are born each year exceeding exposure limits associated with long term IQ deficits. Given these severe effects and high costs to society, information on the benefits of global emissions reduction is critically needed to support regulatory efforts aimed at reducing MeHg exposures.

Quantitative relationships linking changes in global anthropogenic mercury releases to human and biological exposures are still being developed. However, available information suggests aggressive global action to curb emissions is necessary to achieve declines in environmental concentrations. Here we briefly review biological exposures to mercury, environmental sources, and trends in concentrations that could impact human exposure. We conclude by discussing implications for prevention strategies.

### Pathways of exposure

A dominant fraction of human exposure to MeHg is from consuming marine fish. For example, for the United States population an estimated 77% of MeHg exposure is from offshore marine fisheries [2,3]. Many vulnerable populations, particularly in northern regions, consume substantial quantities of marine mammals such as whale and seal that are also high in MeHg [4-6].

Given the importance of marine fish for human and biological exposures, much debate has arisen about the origin of mercury in marine ecosystems and the extent of perturbation by human influences. While some prior research suggested that MeHg in marine fish is naturally occurring (e.g., [7]), recent studies indicate that human impacts on ocean ecosystems are larger than previously thought [8,9]. Environmental concentrations of inorganic mercury drive the pool available for conversion to MeHg and subsequent bioaccumulation in aquatic food chains [10-12]. Many new data sets from ocean ecosystems suggest MeHg production is occurring in ocean seawater [13-19] and thus will be affected by changes in inorganic mercury concentrations in seawater.

Fish and other marine species are also susceptible to the impacts of MeHg. Depew et al. [20] showed reproductive and other sublethal effects occur in fish with tissue residues of MeHg below 0.2 μg/g. By contrast, the tissue residue guideline established by the US EPA for safe consumption by humans is 0.3 μg/g. Dietz et al. [21] showed that mercury concentrations already exceed toxicological thresholds for effects in many species including polar bears, and multiple species of seal, birds and fish from the Arctic. Common loons and songbirds in North America are also thought to be at risk from MeHg exposures [22,23].

MeHg concentrations in biota have increased due to anthropogenic mercury in many regions. Museum feathers from an endangered seabird from the North Pacific (the black footed albatross) showed a sharp increase in MeHg concentrations since 1940 [24]. Dietz et al. [25] analyzed museum samples from marine-food webs in the Arctic and observed that mercury concentrations in began to rise around 1200 and showed a steep increase beginning in the mid-19^th^ century. The authors attributed >90% of this rise to anthropogenic mercury sources. Additional long term monitoring of biological datasets are needed to better quantify these trends and their drivers [26]. These data reinforce that anthropogenic mercury sources have had a substantial impact on human and biological exposures over time.

### Sources of environmental mercury

Anthropogenic mercury emissions result both from intentional uses of mercury and from releases as a byproduct of other activities [27]. At present, coal combustion represents a substantial source of mercury to the environment, and about half of present-day emissions are from Asia [9]. Other sources include industrial processes such as cement production and chlor-alkali production, and uses of mercury in mining applications, including artisanal and small-scale gold mining [28].

Mercury emissions can be deposited on very different spatial scales depending on the chemical form in which they are emitted. Mercury in its elemental, gaseous form (Hg^0^) can remain in the atmosphere for up to a year, transporting globally. Sources that release divalent (Hg^II^) and particulate (Hg^P^) mercury are shorter-lived, resulting in localized deposition. Previous work for the United States showed that while North American sources contribute only an average of 20% to domestic deposition, this fraction rises to 50% at locations downwind of major sources in the industrial Midwest [29].

**Figure 1:**
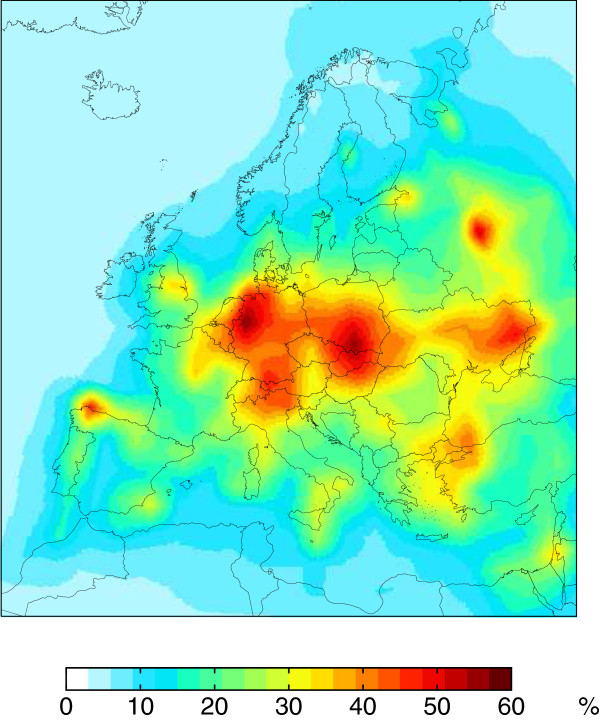
**Percent contribution of European anthropogenic emissions to total (wet plus dry) annual mercury deposition in the GEOS**-**Chem model (v. 9- 01- 03).** Average for meteorological years 2004–2005.

Here, we show a similar analysis for Europe. Figure 1 shows the fraction of present day atmospheric mercury deposition in different regions of Europe from European anthropogenic sources, based on the GEOS-Chem global chemical transport model. European emissions contribute up to 60% of the deposition to ecosystems in industrial areas of Europe. In other areas, such as the Mediterranean, European emissions contribute 20% or less. This suggests that reducing deposition requires local, regional, and global policy actions [30].

Mercury previously released from human activities continues to cycle through the atmosphere, ocean and terrestrial systems for hundreds to thousands of years [31]. This “legacy” mercury is emitted from land and ocean surfaces, and is a substantial component of present-day deposition to ecosystems. Prior work on the global biogeochemical cycle of mercury has estimated that present-day or “primary” anthropogenic emissions contribute about a third of the annual emissions to the atmosphere, with the remaining fractions from legacy and natural mercury sources [12,32-34]. Recent work by Amos et al. [8] suggests that the natural fraction of atmospheric mercury deposition is only 14% and will continue to be diminished over time if anthropogenic emissions continue on their present trajectory [8]. Results from Amos et al. [8] were derived by considering the full history of anthropogenic mercury emissions, including releases prior to the industrial period that are estimated to be 1/3 of all time anthropogenic emissions [9].

### Future trends in mercury concentrations

**Figure 2:**
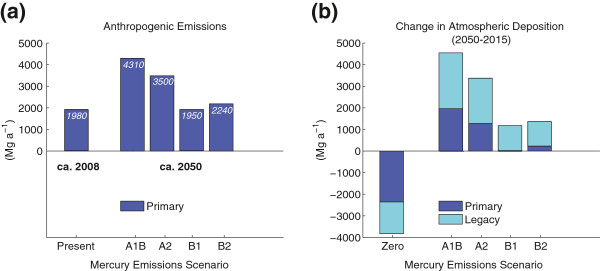
**Projected primary anthropogenic mercury emissions in 2050 (panel a) and resulting changes in atmospheric deposition relative to 2015 (panel b).** Year 2050 mercury emissions scenarios are from Streets et al. [34] based on IPCC projections and adjusted biomass burning for consistency with Streets et al. [9]. These scenarios are bounded by AIB representing a “business-as-usual” scenario with rapid economic growth, while B2 represents a best-case scenario without mercury specific controls (environmental sustainability initiatives and widespread implementation of control technology). The legacy component of deposition is driven by reemissions of previously deposited anthropogenic mercury from oceanic and terrestrial ecosystems. Panel (**b**) is based on the modeling analysis of Amos et al. [8].

Anthropogenic mercury emissions are expected to rise or remain close to constant in the future, mainly because of the expansion of coal combustion projected in Asia [35]. Future mercury emissions projections to 2050 developed by Streets et al. [35] are shown in Figure 2a based on Intergovernmental Panel on Climate Change (IPCC) scenarios. The A1B scenario represents a growth storyline that is roughly equivalent to business-as-usual while the B scenarios represent local (B2) and global (B1) environmental sustainability initiatives. A2 represents large population growth but regionally oriented economic development. Emissions of mercury under B2 are roughly equivalent to present-day emissions levels (i.e., constant emissions from now to 2050). The B scenarios do not consider reductions achievable with mercury specific control technology. Thus, widespread application of mercury specific control technology globally could reduce future deposition to beyond the best-case (B1/B2) scenarios shown in Figure 2a.

Anthropogenic mercury emissions and subsequent deposition to ecosystems will continue to increase the legacy pool. This phenomenon is illustrated by Figure 2b, which shows legacy emissions make up a substantial component of 2050 deposition. The growth of legacy emissions reflects the fully-coupled biogeochemical cycle of mercury, where anthropogenic mercury is released from short and long lived reservoirs in terrestrial ecosystems and the oceans over timescales ranging from months to millennia. Results shown here are adapted from the biogeochemical modeling analysis of Amos et al. [8]. A zero anthropogenic emissions scenario bounds the extent of possible reductions in mercury emissions. Changes in deposition from the legacy pool have largely not been considered by the regulatory community to date.

Considering only changes in primary emissions (Figure 2a) may underestimate the extent of future increases in 2050 deposition (e.g., [36,37]) because such an analysis does not capture changes in the legacy mercury pool. One major benefit of near-term emissions reductions is a decline in the growth rate of the legacy pool that is sustained into the future (e.g., contrast the magnitude of legacy associated deposition in the A1B and B2 scenarios shown in Figure 2b). Decreases in atmospheric deposition on a global scale relative to present will only be possible in the future if emissions reductions are dramatic. Reaching such targets would likely require the application of mercury specific control technologies.

Sunderland et al. [17] used a combination of observations and modelling simulation to infer that at constant emissions levels seawater mercury concentrations in the North Pacific Ocean, a major seafood harvesting region, will rise by 50% in 2050 relative to 1995 levels. These results did not consider the additional burden imposed by the increasing fraction of mercury deposition from legacy emissions, as shown in Figure 2b. Thus, the trajectory of future MeHg concentrations in wild-fish stocks from the Pacific Ocean may be particularly dire in the absence of aggressive global cuts in anthropogenic mercury releases.

### Implications for prevention strategies

Bellanger et al. [1] show that the annual economic costs associated with MeHg exposures in Europe are presently large. These exposures result from a combination of sources, including local and global contributions, as well as influence from present-day and legacy sources. Recent data and modeling studies suggest exposures may increase substantially in the future, particularly for commercial marine seafood consumers, assuming unchanged dietary habits and availability of fish.

From a policy perspective, designing interventions to effectively mitigate the harms posed by mercury requires actions that consider a variety of temporal and spatial scales. Potential interventions include both mitigation measures such as emissions reductions as well as adaptation strategies [30]. Implementation of mercury controls can lead to reductions in fish MeHg concentrations at local scales [10], but global-scale action is also necessary to address the mercury problem [38]. The ongoing negotiations under the United Nations Environment Programme for a global mercury treaty represent a step in that direction.

## Conclusions

The long timescales of mercury cycling through the subsurface and deep ocean mean that any anthropogenic mercury releases persist and can affect biological exposures from timescales of centuries to millennia. As seafood is an important component of a healthy diet for many individuals, short-term solutions to elevated MeHg exposures will require changes in seafood consumption considering both risks and benefits [39]. While concentrations of MeHg in marine fish are likely to continue to exceed threshold levels, emissions reductions will have long-term benefits. Though full ecosystem recovery is only possible in the very distant future, policy action will benefit vulnerable species that accumulate MeHg and prevent further increases in MeHg in consumed species.

## Abbreviations

EPA: Environmental Protection Agency; EU: European Union; Hg^0^: elemental mercury; Hg^II^: divalent inorganic mercury; Hg^P^: particulate mercury; MeHg: Methylmercury; IPCC: Intergovernmental Panel on Climate Change; IQ: Intelligence Quotient; US: United States.

## Competing interests

The authors declare they have no competing interests.

## Authors’ contributions

EMS initiated the manuscript. EMS and NES both drafted the manuscript, read and approved the final version.
